# Quantifying population-level health benefits and harms of e-cigarette use in the United States

**DOI:** 10.1371/journal.pone.0193328

**Published:** 2018-03-14

**Authors:** Samir S. Soneji, Hai-Yen Sung, Brian A. Primack, John P. Pierce, James D. Sargent

**Affiliations:** 1 Norris Cotton Cancer Center, Geisel School of Medicine at Dartmouth, Lebanon, NH, United States of America; 2 Dartmouth Institute for Health Policy & Clinical Practice, Geisel School of Medicine at Dartmouth, Lebanon, NH, United States of America; 3 Institute for Health & Aging, School of Nursing, University of California, San Francisco, San Francisco, CA, United States of America; 4 Division of General Internal Medicine, Department of Medicine, School of Medicine, University of Pittsburgh, Pittsburgh, PA, United States of America; 5 Moores Cancer Center, University of California, San Diego, San Diego, CA, United States of America; 6 Department of Family Medicine & Public Health, University of California, San Diego, San Diego, CA, United States of America; University of Montana, UNITED STATES

## Abstract

**Background:**

Electronic cigarettes (e-cigarettes) may help cigarette smokers quit smoking, yet they may also facilitate cigarette smoking for never-smokers. We quantify the balance of health benefits and harms associated with e-cigarette use at the population level.

**Methods and findings:**

Monte Carlo stochastic simulation model. Model parameters were drawn from census counts, national health and tobacco use surveys, and published literature. We calculate the expected years of life gained or lost from the impact of e-cigarette use on smoking cessation among current smokers and transition to long-term cigarette smoking among never smokers for the 2014 US population cohort.

**Results:**

The model estimated that 2,070 additional current cigarette smoking adults aged 25–69 (95% CI: -42,900 to 46,200) would quit smoking in 2015 and remain continually abstinent from smoking for ≥7 years through the use of e-cigarettes in 2014. The model also estimated 168,000 additional never-cigarette smoking adolescents aged 12–17 and young adults aged 18–29 (95% CI: 114,000 to 229,000), would initiate cigarette smoking in 2015 and eventually become daily cigarette smokers at age 35–39 through the use of e-cigarettes in 2014. Overall, the model estimated that e-cigarette use in 2014 would lead to 1,510,000 years of life lost (95% CI: 920,000 to 2,160,000), assuming an optimistic 95% relative harm reduction of e-cigarette use compared to cigarette smoking. As the relative harm reduction decreased, the model estimated a greater number of years of life lost. For example, the model estimated-1,550,000 years of life lost (95% CI: -2,200,000 to -980,000) assuming an approximately 75% relative harm reduction and -1,600,000 years of life lost (95% CI: -2,290,000 to -1,030,000) assuming an approximately 50% relative harm reduction.

**Conclusions:**

Based on the existing scientific evidence related to e-cigarettes and optimistic assumptions about the relative harm of e-cigarette use compared to cigarette smoking, e-cigarette use currently represents more population-level harm than benefit. Effective national, state, and local efforts are needed to reduce e-cigarette use among youth and young adults if e-cigarettes are to confer a net population-level benefit in the future.

## Introduction

The use of electronic cigarettes (e-cigarettes) has become intensely controversial since their introduction to the US in 2007 [1–7]. E-cigarettes might help the 40 million current adult cigarette smokers quit—the vast majority of whom want to stop smoking completely—by delivering nicotine with the same sensory experience as combustible, or traditional, cigarettes but without inhalation of as many toxicants [8–12]. Conversely, e-cigarettes might facilitate the transition to traditional cigarette smoking among never-smoking adolescents and young adults [13–21]. This harm is potentially substantial because youth e-cigarette use has risen rapidly over time [6,22,23]. For example, past 30-day use of e-cigarettes increased from 1.5% in 2011 to 11.3% in 2016 among high school students and exceeded their level of past 30-day use of traditional cigarettes (8.0% in 2016) [24].

The controversy over e-cigarettes persists because we do not yet know if e-cigarette use results in more benefit than harm at the population level [25–27]. This uncertainty creates a quandary for the US Food and Drug Administration (FDA), which recently asserted its regulatory authority over e-cigarettes and developed regulations to promote their safety and limit youth appeal [28]. Quantifying the balance of benefits and harms of e-cigarette use requires simultaneous accounting of the additional number of (1) current cigarette smokers who will quit through the use of e-cigarettes and (2) never-cigarette smokers who will initiate cigarette smoking through the use of e-cigarettes, a substantial proportion of whom may become long-term daily cigarette smokers. A recent study concluded a net population-level health benefit under a scenario in which e-cigarette use increases in the future only among cigarette smokers interested in quitting, and net harm under a scenario in which e-cigarette use increases in the future only among youth who would have never smoked [29]. A second study modeled future cigarette and e-cigarette use patterns over the next decade for young adults aged 18–24 years and concluded that e-cigarette use would have a limited impact on the prevalence of current cigarette smoking [30]. However, this study did not assess the effect of e-cigarette use among adolescents or adults aged ≥25 years. A third study estimated the population impact of e-cigarettes on smoking cessation and found e-cigarettes could increase the number of smokers who successfully quit for one year. However, this study also did not assess the effect of e-cigarette use among adolescents [31]. Thus, these last two studies could not determine the balance of benefits and harms of e-cigarette use at the population level.

In this study, we developed a Monte Carlo stochastic simulation model that extends prior research in two ways. First, we simultaneously consider multiple population subgroups including current cigarette smokers and never cigarette smokers. Second, we quantify the net population benefits (or harms) of e-cigarette use in terms of the total number of years of life gained among additional current cigarette smokers who quit smoking and years of life lost among additional cigarette smoking initiators who become long-term daily cigarette smokers, both through the use of e-cigarettes. We base our calculations on 2014 US census data, national health or tobacco use surveys on e-cigarette use, and published randomized trials and cohort studies on the e-cigarette associated transition probabilities of cigarette smoking cessation and initiation.

## Methods

### Analytic model

**Fig 1:**
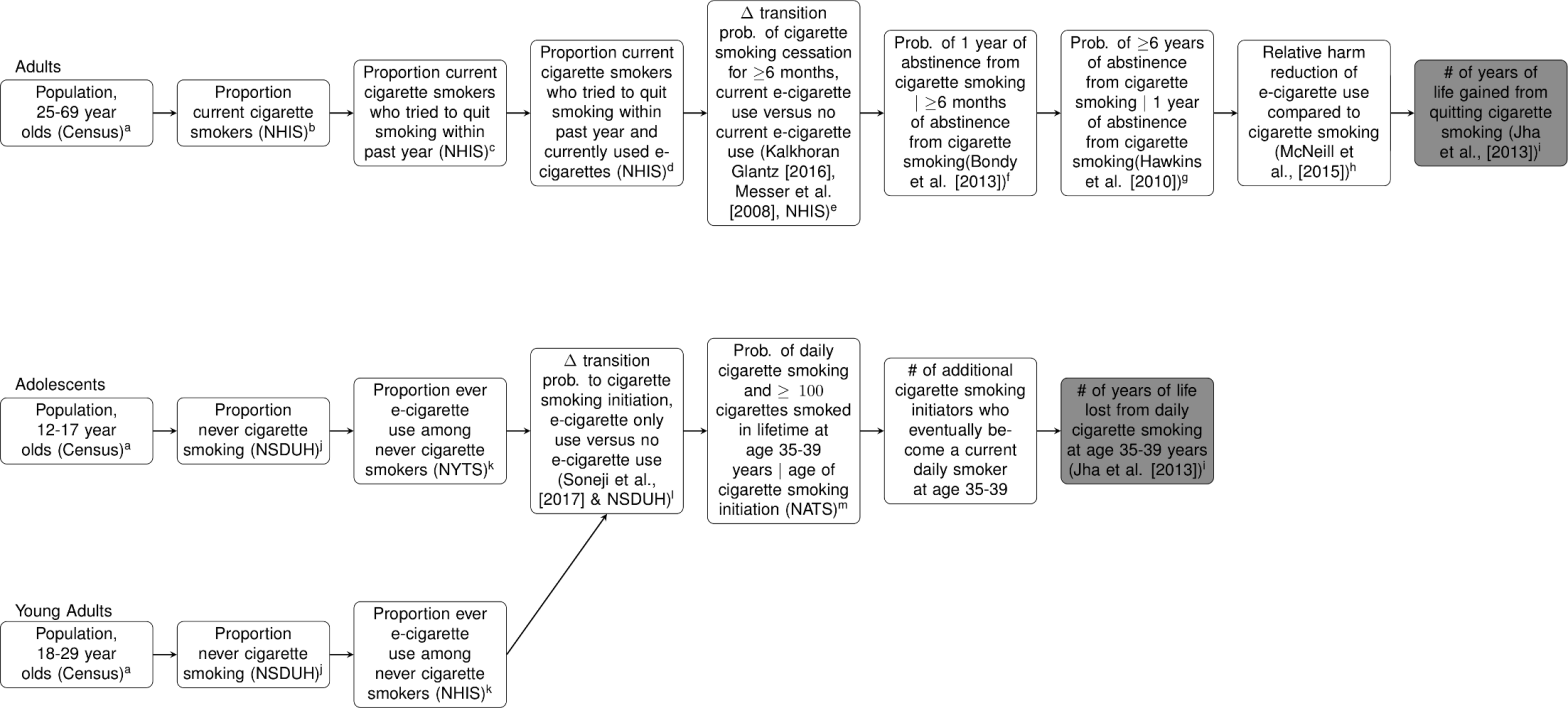
Population-level model to quantify benefits and harms of E-cigarette use. Superscripted letters refer to the columns in Tables A and B in S3 Appendix for age- and age-group-specific parameter point estimates and 95% confidence intervals. Note: Δ = Change in; | = Conditional On; NATS = National Adult Tobacco Survey; NHIS = National Health Interview Survey; NSDUH = National Survey on Drug Use and Health; NYTS = National Youth Tobacco Survey; and Prob. = Probability.

Our analytic approach consists of two main steps (Fig 1). The first step estimates the number of years of life gained among the additional number of current cigarette smokers who quit smoking through the use of e-cigarettes as a cessation tool, compared to those who did not use e-cigarettes as a cessation tool, and remain continually abstinent from smoking for ≥7 years. We set the threshold for continual abstinence at 7 years because cohort studies found that relapse beyond this point is rare [32,33]. Additionally, the risk of death among former cigarette smokers who quit for this long begins to approximate the risk of death among never cigarette smokers [34]. We began with the US adult population of 25–69 year olds in 2014 (in five-year age groups) and multiplied these counts by the: (1) age-group-specific prevalence of current cigarette smoking, (2) age-group-specific prevalence of trying to quit smoking within the past year among current cigarette smokers, (3) age-group-specific prevalence of current e-cigarette use among current cigarette smokers who tried quitting within the past year, (4) difference in the transition probability of ≥6-month cigarette smoking cessation between current smokers who used e-cigarettes as a cessation tool and current smokers who did not use e-cigarettes as a cessation tool, (5) probability of 1 year of cigarette smoking abstinence from cigarette smoking given ≥6 months of cigarette smoking abstinence, (6) probability of ≥6 years of abstinence from cigarette smoking given 1 year of cigarette smoking abstinence, and (7) age-group-specific number of years of life gained from quitting cigarette smoking. We assumed 95% relative harm reduction of e-cigarette use, compared to cigarette smoking, among current cigarette smokers who used e-cigarettes as a cessation tool and quit smoking [35]. As described below, we vary the relative harm of e-cigarette use, compared to cigarette smoking, to include the levels of relative harm inferred from in vitro and mouse model studies [36,37].

The second step estimates the number of years of life lost among the additional number of never-cigarette smoking adolescents and young adults who eventually become current daily cigarette smokers (and also smoked ≥100 cigarettes in lifetime) at age 35–39 through the use of e-cigarettes. We began with the US adolescent and young adult population of 12–29 year olds in 2014 (by single year of age) and multiplied these counts by the: (1) age-specific prevalence of never cigarette smoking, (2) age-specific prevalence of ever having tried e-cigarettes among never cigarette smokers, (3) the difference in the transition probability of cigarette smoking initiation among never cigarette smoking adolescents and young adults who had ever used e-cigarettes, compared to the corresponding probability among those who had never used e-cigarettes, (4) probability of becoming a current daily cigarette smoker at age 35–39 based on the age of cigarette smoking initiation, and (5) age-specific number of years of life lost from current daily cigarette smoking at age 35–39.

We assessed three outcomes of interest: (1) the additional number of current cigarette smokers who will quit smoking through the current use of e-cigarettes and abstain from smoking for ≥7 years, compared to those who do not currently use e-cigarettes and (2) the additional number of adolescents and young adults who will initiate cigarette smoking through the ever use of e-cigarettes and eventually become daily cigarette smokers at age 35–39, compared to those who never used e-cigarettes; and (3) the total number of expected years of life gained or lost across all these population subgroups.

Table 1 describes the data source of each model parameter. S1 Appendix describes how the difference in transition probabilities of ≥6-month cigarette smoking cessation between current e-cigarette users and non-current e-cigarette users was estimated based on various parameters such as the proportion of current cigarette smokers who used pharmaceutical aids during quit attempt and the pooled odds ratio of quitting smoking among smokers interested in quitting reported by the meta-analysis of Kalkhoran & Glantz [38]. S2 Appendix describes the estimation of the difference in transition probabilities of cigarette smoking initiation between never cigarette smokers who ever used e-cigarettes compared to those who never used e-cigarettes based on the pooled odds ratio of cigarette smoking initiation reported by the meta-analysis of Soneji et al. [19]. Tables A and B in S3 Appendix show the value of each model parameter.

**Table 1:** Data Sources of model parameters.

Parameter	Population Sub-group	Source	Survey Question & Notes
Population	All	2014 US Census	—
Current Cigarette Smoking	Current Smokers	2014 NHIS	“Have you smoked at least 100 cigarettes in your entire life?” (yes). “Do you now smoke cigarettes every day, some days or not at all?” (every day or some days)
Past-Year Quit Attempt	Current Smokers	2014 NHIS	“During the past 12 months, have you stopped smoking for more than one day because you were trying to quit smoking?” (yes)
Current E-Cigarette Use	Current Smokers	2014 NHIS	“Do you now use e-cigarettes every day, some days, or not al all?” (every day or some days)
Proportion Of Current Cigarette Smokers With a Past-Year Quit Attempt Who Used a Pharmaceutical Aid During Quit Attempt* (%)	Current Smokers	2010 NHIS	“Thinking back to when you tried to quit smoking in the past 12 months, did you use any of the following products: a nicotine patch; a nicotine gum or lozenge; a prescription pill, such as Zyban, Bupropion, or Wellbutrin; a nicotine containing nasal spray or inhaler; a nicotine patch?”. See S1 Appendix for calculation of e-cigarette associated Δtransition probability of ≥6-months cigarette smoking cessation.
Probability of Cigarette Smoking Cessation ≥6 Months Among Current Cigarette Smokers Who Seriously Tried to Quit and Used a Pharmaceutical Aid During Quit Attempt (%)	Current Smokers	Messer et al. [92]	“Thinking back to the last time you tried to quit smoking in the past 12 months. Did you use any of the following products: a nicotine gum; a nicotine patch; a nicotine nasal spray; a nicotine inhaler; a nicotine lozenge; a nicotine tablet; a prescription pill, such as Zyban, Buproprion, or Wellbutrin?” (2003 TUS-CPS). “During the past 12 months, what is the length of time you stopped smoking because you were trying to quit smoking?” (2003 TUS-CPS). See S1 Appendix for calculation of e-cigarette associated Δtransition probability of ≥6-months cigarette smoking cessation.
Odds Ratio of Quitting Smoking Among Smokers with an Interest in Quitting	Current Smokers	Kalkhoran & Glantz [38]	Meta-analysis of 2 clinical trials [49,93], 4 cohort studies [50,51,63,94], and 1 cross-sectional study [52]. See S1 Appendix for calculation of e-cigarette associated transition probability of ≥6-months cigarette smoking cessation
Relative Risk Of Cigarette Smoking Cessation Among Current Cigarette Smokers Interested In Quitting, E-Cigarette Users Compared With Nicotine Patch Users	Current Smokers	Bullen et al. [49]	Primary outcome was continuous ≥6-month smoking abstinence: self-reported abstinence over the whole follow-up period (allowing ≤5 cigarettes in total) and biochemically verified continuous abstinence at 6 months (exhaled breath carbon monoxide measurement <10 ppm). See S1 Appendix for calculation of e-cigarette associated transition probability of ≥6-months cigarette smoking cessation.
Probability of 1-Year Abstinence from Cigarette Smoking | 6-Months Abstinence	Current Smokers	Bondy et al. [95]	2005–2008 Ontario Tobacco Survey
Probability of Long-Term (≥6-Year) Abstinence from Cigarette Smoking | ≥1-Year Abstinence	Current Smokers	Hawkins et al. [33]	1991–2006 British Household Panel Survey
Relative Harm Reduction of E-Cigarette Use Compared to Cigarette Smoking	Current Smokers	McNeill et al. [35]	Consensus opinion
Never Cigarette Smoking	Adol. & Young Adults	2014 NSDUH	“Have you ever tried cigarette smoking, even one or two puffs?” (no)
Ever E-Cigarette Use	Adol.	2014 NYTS	“Have you ever used an electronic cigarette, even just one time in your entire life?” (yes)
Ever E-Cigarette Use	Young Adults	2014 NHIS	“Have you ever used an electronic cigarette, even just one time in your entire life?” (yes)
Probability of Cigarette Smoking Initiation Among Never E-Cigarette Users	Adol. & Young Adults	2012 Surgeon General’s Report [96]	Initiation of cigarette smoking 12- to 17-year-olds and 18- to 25-year olds, 2006 (2006–2010 NSDUH). See S2 Appendix for calculation of e-cigarette associated transition probability of cigarette smoking initiation.
Adjusted Odds Ratio of Cigarette Smoking Initiation, Ever E-Cigarette Users vs. Never E-Cigarette Users	Adol. & Young Adults	Soneji et al. (2017)[19]	Seven cohort studies pooled in random-effects meta-analysis [13–18,97]. Odds ratio—adjusted for demographic, psychosocial, and behavioral risk factors—of cigarette smoking initiation between never cigarette smokers who ever used e-cigarettes and never cigarette smokers who never used e-cigarettes. See S2 Appendix for calculation of e-cigarette associated Δtransition probability of cigarette smoking initiation.
Probability of Being a Current Daily Cigarette Smoker at Age 35–39 | Age Of Cigarette Smoking Initiation	Adol. & Young Adults	2009–2010 and 2012–2013 NATS	Current daily cigarette smoker at age 35–39: “Have you smoked at least 100 cigarettes in your entire life?” (yes). “Do you now smoke cigarettes every day, some days, or not at all?” (every day or some days). Age of cigarette smoking initiation: “How old were you when you smoked a whole cigarette for the first time?”
Years of Life Gained or Lost	All	Jha et al.[98]	1997–2004 NHIS data linked to National Death Index. Years of life gained applied to current cigarette smokers who quit for ≥6 years. Years of life lost applied to adolescents and young adults who become current daily cigarette smokers at age 35–39.

Note: Adol. = Adolescents; | = Conditional On; NATS = National Adult Tobacco Survey; NHIS = National Health Interview Survey; NSDUH = National Survey on Drug Use and Health; NYTS = National Youth Tobacco Survey; TUS-CPS = Tobacco Use Supplement, Current Population Survey.

### Validation of model

We validated the model against one-year intermediate outcomes (e.g., the number of adolescents and young adult cigarette smoking initiators). For current adult smokers, we applied the model to 2013 National Health Interview Survey (NHIS) data to predict the number of current cigarette smoking adults (both current and non-current e-cigarette users) who would quit in 2014 and remain continually abstinent from smoking for ≥6 months. We then compared this predicted number with the observed number in 2014, estimated from 2014 NHIS data, by identifying new ≥6-month quitters as respondents who answered six months to one year to the question: “How long has it been since you quit smoking cigarettes?”. For adolescent and young adult never smokers, we applied the model to 2013 National Survey on Drug Use and Health (NSDUH) data to predict the number of cigarette smoking initiators in 2014 (both ever and never e-cigarette users). We then compared this predicted number with the observed number of initiators in 2014, estimated from 2014 NSDUH data, by identifying respondents who answered “yes” to the question: “Have you smoked part or all of a cigarette?” and whose current age was ≤1 year less than the age at which they first smoked a cigarette (“How old were you the first time you smoked part or all of a cigarette?”).

### Analytic considerations and sensitivity analyses

To account for uncertainty in the prevalence and transition probability parameters, we utilized Monte Carlo simulation and independently drew from normal distributions with the means and standard deviations equal to the parameters’ means and standard errors shown in Tables A and B in S3 Appendix. We repeated this process 100,000 times to create a distribution of each outcome of interest.

We conducted a sensitivity analysis by varying the level of four key parameters: (1) the adjusted odds ratio of smoking cessation, (2) the adjusted odds ratio of cigarette smoking initiation, (3) age-group-specific prevalence of current e-cigarette use among current cigarette smokers who tried quitting within the past year, and (4) age-specific prevalence of ever having tried e-cigarettes among never cigarette smokers. We also calculated the probability of positive total years of life gained across a wide range of possible values for these four parameters. For example, we supposed the adjusted odds ratio of smoking cessation equaled 2.5 times the baseline estimate (2.15 = 2.5 x 0.86) and recalculated the years of life gained, drawing all other parameters from their baseline distributions. The probability of a positive total years of life gained under this supposition equaled the ratio of the (1) number of simulations that yielded a positive value and (2) total number of simulations (100,000). Finally, we varied from 0% to 100% the relative harm of e-cigarette use, compared to cigarette smoking, in terms of the number of years of life gained from quitting cigarette smoking. We used R, Version 3.2.3 for all analyses. Results of years of life gained were determined to be statistical significant if their 95% confidence intervals do not contain zero.

## Results

### Additional quitters and initiators

In 2014, 3,490,000 current adult cigarette smokers who had attempted to quit smoking in the past year had also currently used e-cigarettes. Additionally, 3,640,000 never-cigarette smoking adolescents and young adults had ever used e-cigarettes.

**Fig 2:**
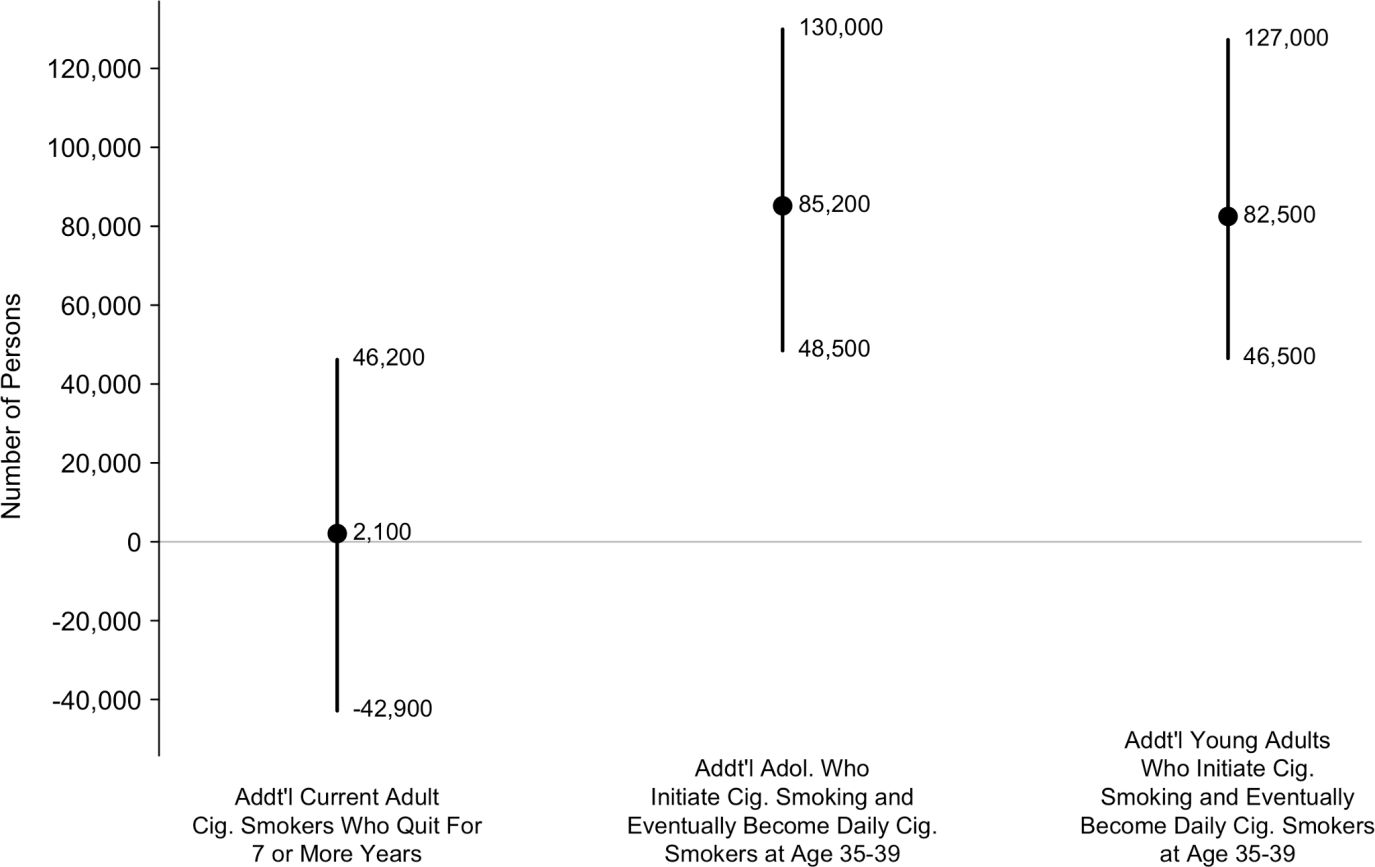
Number of additional adult current cigarette smokers who quit for ≥7 years and additional adolescents and young adults who initiate cigarette smoking and eventually become daily cigarette smokers at age 35–39, all through the use of E-cigarettes. The mean of the distribution is shown as a solid circle and the 95% confidence interval is shown as a vertical line. Source: stochastic simulation (100,000 iterations). Note: Addt’l = Additional; Cig. = Cigarette. Estimates reported as text in the figure rounded to 3 significant digits.

The model estimated that 2,070 additional current cigarette smoking adults (95% CI: -42,900 to 46,200) who currently used e-cigarettes in 2014 would quit smoking in 2015 and remain continually abstinent from smoking for ≥7 years using e-cigarettes, compared to those who did not currently use e-cigarettes (Fig 2). The model also estimated that an additional 168,000 never-cigarette smoking adolescents and young adults in 2014 (95% CI: 114,000 to 229,000) who had ever used e-cigarettes would initiate cigarette smoking in 2015 and eventually become daily cigarette smokers at age 35–39, compared to those who had never used e-cigarettes.

### Years of life gained

**Fig 3:**
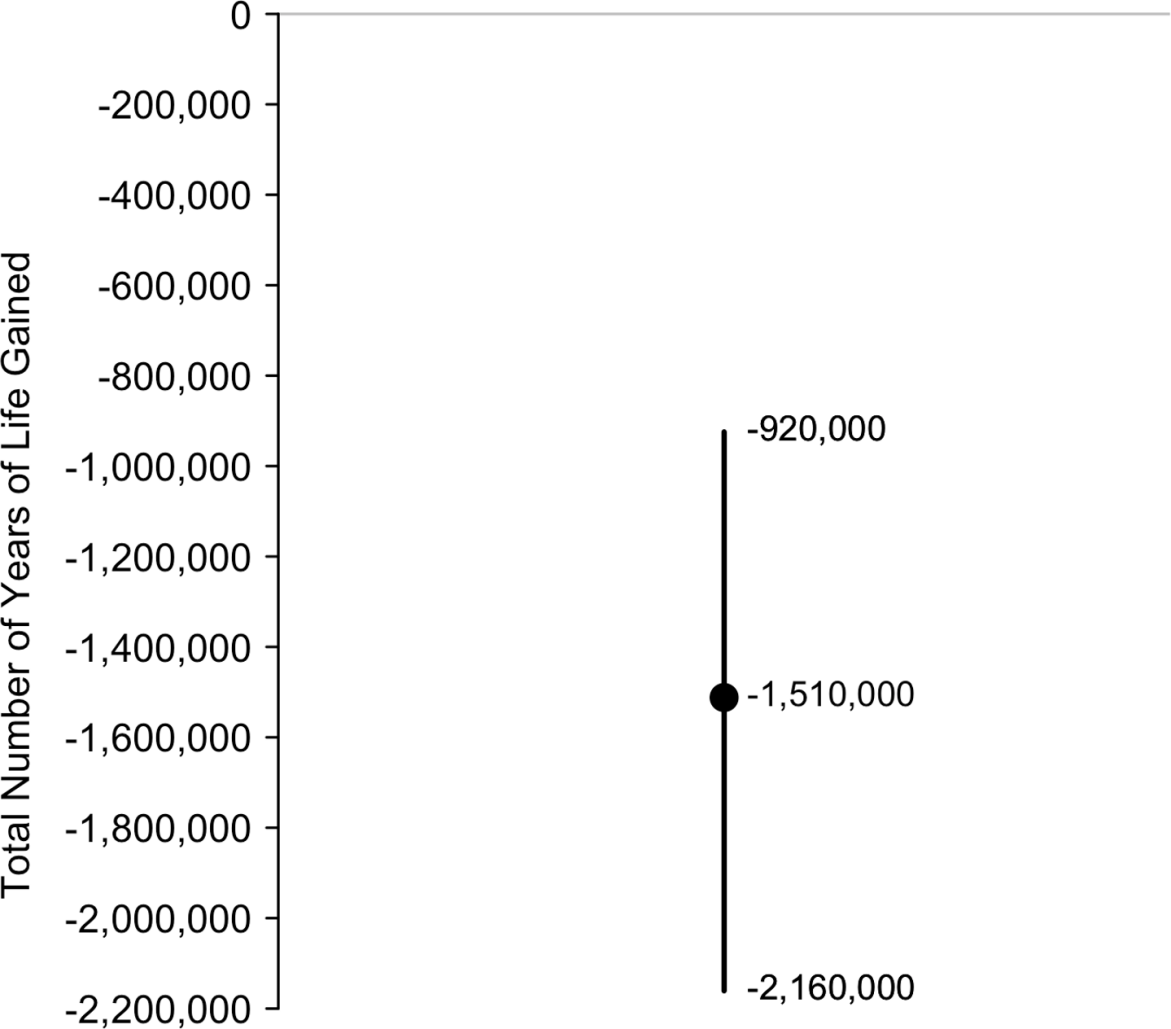
Total number of years of life gained. Negative years of life gained represent years of life lost. The mean of the distribution is shown as a solid circle and the 95% confidence interval is shown as a vertical line. Source: stochastic simulation (100,000 iterations). Estimates reported as text in the figure rounded to 3 significant digits.

The model estimated that the 2,070 additional long-term quitters would gain -3,000 years of life (95% CI: -351,000 to 325,000). The model also estimated the additional 168,000 adolescent and young adult cigarette smoking initiators who eventually become daily cigarette smokers at age 35–39 will lose 1,510,000 years of life (95% CI: 1,030,000 to 2,060,000). Thus, considering all population subgroups, the model estimated that e-cigarette use in 2014 would lead to 1,510,000 years of life lost (95% CI: 920,000 to 2,160,000; Fig 3) assuming an approximate 95% relative harm reduction of e-cigarette use compared to cigarette smoking.

### Sensitivity analysis

**Fig 4:**
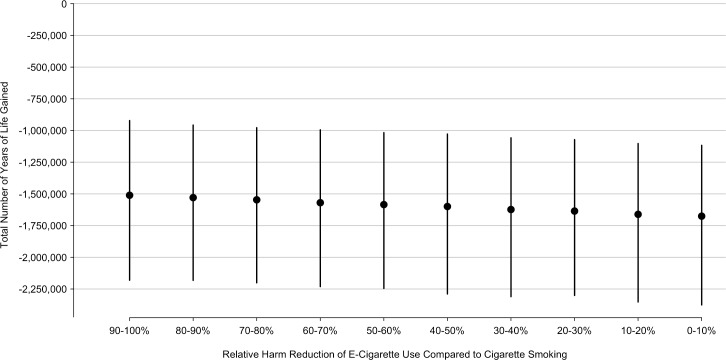
Total number of years of life gained by relative harm of E-cigarette use compared to cigarette smoking.

Our results were sensitive to the adjusted odds ratios of cigarette smoking cessation and cigarette smoking initiation (Table 2). The model estimated that e-cigarette use in 2014 would lead to 1,150,000 years of life lost (95% CI: 2,130,000 to 242,000) under the relative risk of smoking cessation estimated by Bullen et al. (transformed to an odds ratio). The model estimated that e-cigarette use in 2014 would lead to 1,330,000 years of life lost (95% CI: 1,950,000 to 780,000) and 1,150,000 years of life lost (95% CI: 1,730,000 to 620,000) if the adjusted odds ratio of cigarette smoking initiation decreased by 10% and 20%, respectively. Our results were also sensitive to the prevalence of current e-cigarette use among current cigarette smokers who tried quitting within the past year and ever e-cigarette use and never cigarette smokers. Finally, we varied the health risks of e-cigarette use as a percentage of the risk associated with cigarette smoking. The total number of years of life lost increased as the relative harm of e-cigarette use, compared to cigarette smoking, grew (Fig 4). The model estimated that e-cigarette use in 2014 would lead to 1,530,000 years of life lost (95% CI: 2,180,000 to 960,000) and 1,580,000 years of life lost (95% CI: 2,250,000 to 1,020,000) if the health risks of e-cigarette use were 10%-20% (i.e., 80%-90% safer) and 40%-50% (i.e., 50%-60% safer) of the risks of cigarette smoking, respectively.

**Table 2:** Results of sensitivity analysis.

Parameter	Scenario	Parameter Pt. Est. (95% CI)	Years of Life Gained (95% CI)^2^
Adjusted Odds Ratio of Cigarette Smoking Cessation	Base Case	0.86 (0.54 to 1.18)	-1,510,000 (-2,160,000 to -925,000)
	Bullen et al.^1^	1.28 (0.42 to 2.24)	-1,150,000 (-2,130,000 to -242,000)
Adjusted Odds Ratio of Cigarette Smoking Initiation	Base Case	3.50 (2.38 to 5.16)	-1,510,000 (-2,160,000 to -925,000)
	10% Reduction	3.15 (2.14 to 4.64)	-1,330,000 (-1,950,000 to -775,000)
	20% Reduction	2.80 (1.90 to 4.13)	-1,150,000 (-1,730,000 to -616,000)
Prevalence of Current E-Cigarette Use Among Current Cigarette Smokers Who Tried to Quit Within the Past Year	Base Case	Age-Group Specific	-1,510,000 (-2,160,000 to -925,000)
	10% Increase	Age-Group Specific	-1,510,000 (-2,180,000 to -906,000)
	20% Increase	Age-Group Specific	-1,510,000 (-2,190,000 to -882,000)
Prevalence of Ever E-Cigarette Use Among Never Cigarette Smokers	Base Case	Age Specific	-1,510,000 (-2,160,000 to -925,000)
	10% Decrease	Age Specific	-1,360,000 (-1,950,000 to -817,000)
	20% Decrease	Age Specific	-1,210,000 (-1,770,000 to -702,000)

Note: Pt. Est. = Point Estimate; CI = Confidence Interval.

^1^Odds ratio and 95% CI converted from reported relative risk and probability of 6-month cessation in the nicotine patch control group (5.8%).

^2^All estimates rounded to 3 significant digits.

**Fig 5:**
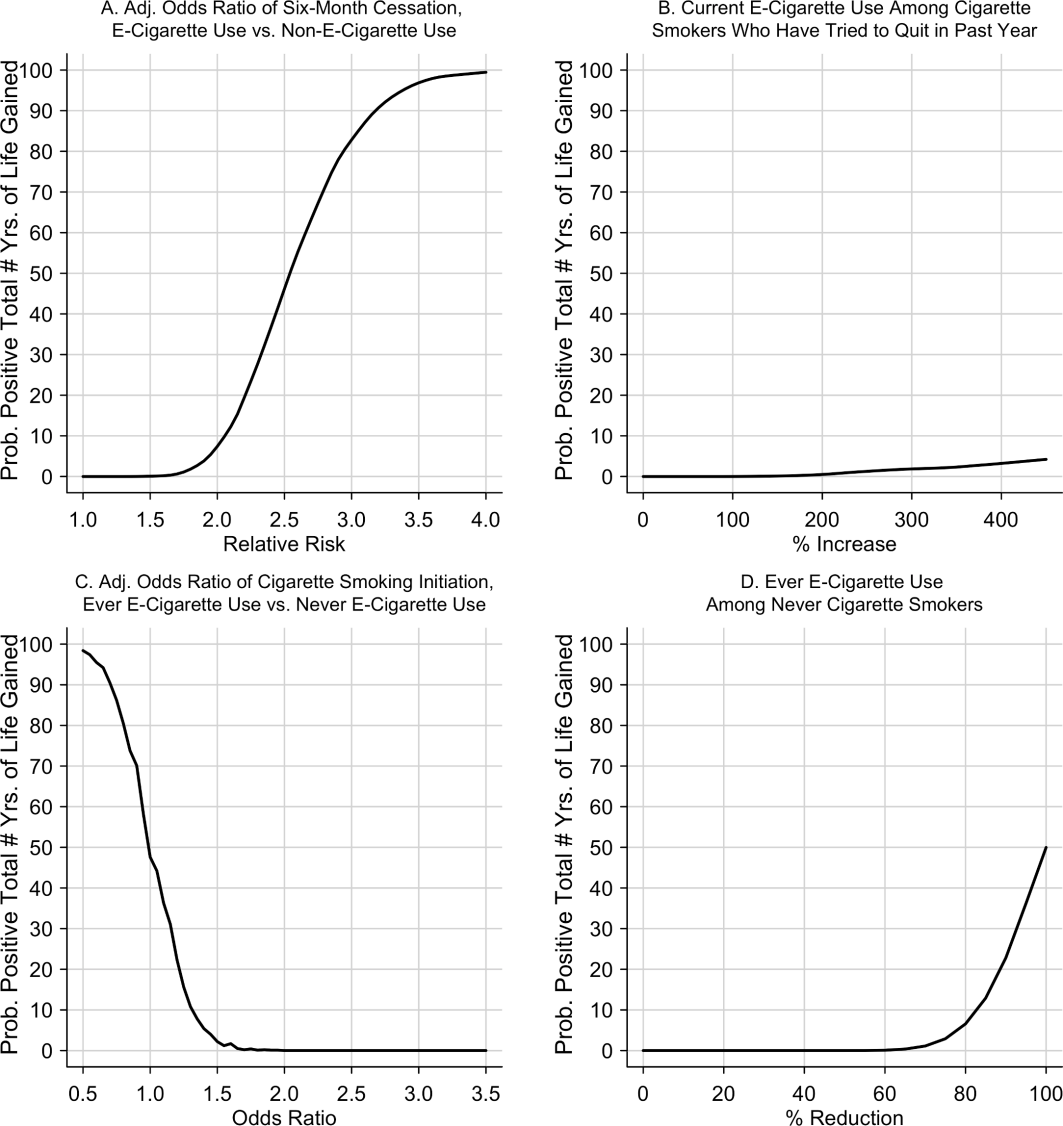
Probability of a positive total number of years of life gained varying the level of four key model parameters. Note: vs. = versus; Adj. = Adjusted.

The probability of a positive total number of years of life gained increased with the relative risk of smoking cessation: 6.7%, 44.6%, and 83.3% as the relative risk increased to 2.0, 2.5, and 3.0, respectively (Fig 5, Panel A). The probability also increased with higher prevalence of current e-cigarette use among current cigarette smokers (Fig 5, Panel B). Conversely, the probability increased to 0.0%, 0.0%, and 47.6% as the adjusted odds ratio decreased to 3.0, 2.0, and 1.0, respectively (Fig 5, Panel C). Finally, the probability increased with lower prevalence of ever e-cigarette use among never cigarette smokers (Fig 5, Panel D).

### Model validation

Based on 2013 NHIS data, we predicted 1.2 million current cigarette smoking adults would have quit and remained continually abstinent from smoking for ≥6 months in 2014 (95% CI, 1.0 to 1.4 million), which was not statistically different (p = 0.57) from the estimated number from the 2014 NHIS data (1.1 million, 95% CI: 0.9 to 1.3 million). Based on 2013 NSDUH data, we predicted that 5.5 million adolescents and young adults would have initiated cigarette smoking in 2014 (95% CI: 4.0 to 6.9 million), which was not statistically different (p = 0.53) from the observed number from 2014 NSDUH data (5.0 million, 95% CI: 4.1 to 5.9 million).

## Discussion

Our study developed a Monte Carlo stochastic simulation model to assess the balance of health benefits and harms of e-cigarette use at the population level. Based on the most up-to-date published evidence, our model estimated that e-cigarette use in 2014 represents a population-level harm of about 1.6 million years of life lost over the lifetime of all adolescent and young adult never-cigarette smokers and adult current cigarette smokers in the 2014 US population. Our model also estimated even greater population-level harm if e-cigarette use confers long-term health risks.

Our study is consistent with Kalkhoran & Glantz (2015), who estimated the effects of e-cigarette use on cessation among smokers and on cigarette smoking initiation by never-smokers under various scenarios [29]. For example, their study found the largest relative health costs occurred in the scenario under which e-cigarette use increased among never-smokers because of the resulting increase in cigarette smoking initiation and the dual use of cigarettes and e-cigarettes, while e-cigarette use remained unchanged among established smokers. Our study also supports the conclusion of Cherng et al. (2016) on the relative effects of e-cigarettes on smoking initiation and cessation [39]. Our model indicates that the odds of smoking initiation among e-cigarette users would need to decrease more than the odds of smoking cessation would need to increase to achieve the same change in the total number of years of life gained.

Our conclusions differ from those of Levy et al. (2016), Levy et al. (2017), and Hill & Camacho (2017)—a tobacco industry-funded study [40–42]. Hill & Camacho found the use of e-cigarettes would result in a decrease in smoking-related mortality in the UK from 8.4% to 8.1% in 2050 [40]. Levy et al. found that the use of vaporized nicotine products (VNPs; e.g., e-cigarettes) would lead to years of life gained for the US birth cohort of 1997 as it ages over time [41]. Hill & Camacho estimated an “overall beneficial effect from launching e-cigarettes”, in part, because they explicitly assumed the transition probability of cigarette smoking initiation among never cigarette smokers who used e-cigarettes equaled 5% [40]. Levy et al. (2016) estimated a “positive public health impact” from VNP use, in part, because they implicitly assumed the odds of cigarette smoking initiation was only marginally higher for ever e-cigarette users than never e-cigarette users (odds ratio≈1.16) among adolescents and young adults who would not have become a cigarette smoker in the absence of VNPs. Yet, both of these assumptions are substantially different from empirical estimates of these parameters from thirteen published cohort studies with a combined sample size of over 44,000 respondents [13–18,20,21,43–47]. Levy et al. (2017) estimates a substantial number of years of life gained from e-cigarette use, in part, because they explicitly assumed e-cigarette use among never cigarette smokers does not increase the rate of cigarette smoking initiation, which—again—contrasts with growing scientific evidence to the contrary. Nevertheless, these models provide useful conceptual frameworks to assess the net benefits of e-cigarette use and would likely yield substantively different conclusions under alternative—and empirically based—assumptions of e-cigarette use and cigarette smoking initiation.

E-cigarettes could, indeed, confer a positive population benefit if they were more effective as a smoking cessation device. For example, if current smokers who used e-cigarettes as a smoking cessation tool achieved six-month smoking abstinence at a rate of approximately 2.55 times greater than their counterparts who did not use e-cigarettes, then our model estimated that the probability of a positive total number of years of life gained would approach 50%. However, the estimated effectiveness of e-cigarettes for smoking cessation from all published randomized trials and nearly all cohort studies fall well below this threshold including some studies that concluded cigarette smokers who used e-cigarettes were less—not more—likely to quit than those who used standard clinic-based smoking cessation treatments [11,38,48–65]. Three cohort studies of current cigarette smokers did, indeed, estimate relative risks of smoking cessation above this threshold among intensive e-cigarette users (daily use for at least one month), daily tank e-cigarette users, and long-term (i.e., ≥2-year) e-cigarette users [59,66,67]. However, the prevalence of intensive e-cigarette use, daily e-cigarette tank use, and long-term e-cigarette use were low in these studies: only 34% of e-cigarette users were intensive users, 12% of e-cigarette users were daily e-cigarette tank users, and 14% of e-cigarette users were long-term users [59,66,67].

A decline in public acceptability of cigarette smoking has been accompanied by proscriptions on where smoking is allowed [68,69]. Nearly two-thirds of e-cigarette users reported using them when and where cigarette smoking was not allowed [70,71]. Further, an analysis of e-cigarette tweets highlighted that e-cigarette vaping was considered social acceptable by many, as opposed to cigarette smoking [72]. However, the lower level of sensation and satisfaction experienced with e-cigarettes, compared to cigarettes, may explain why some individuals who initiate with e-cigarettes then transition to cigarettes even thought this transition is associated with higher nicotine ingestion [73–75].

E-cigarette use among former cigarette smokers may confer health risks. For example, e-cigarette aerosols carry high levels of aldehydes (e.g., formaldehyde) that affect cardiovascular function and high levels of fine particles that accelerate heart disease [76,77]. E-cigarette users experience equivalent reductions in vascular function (e.g., vitamin E levels and flow-mediation dilatation) as cigarette smokers. Furthermore, e-cigarette use suppresses immune and inflammatory-response genes in nasal epithelial cells and injures lung epithelial cells [78,79].

Our study has some potential limitations. First, we do not know if e-cigarette use causes cigarette-smoking initiation in adolescents and young adults. Published cohort studies have found consistent evidence of an increased risk of cigarette smoking initiation among non-smoking youth who had ever used e-cigarettes after accounting for known demographic, psychosocial, and behavioral risk factors [13–18,20,21]. We varied this longitudinal association between e-cigarette use and cigarette smoking initiation and reach similar conclusions. Perhaps more concerning that cigarette smoking initiation, e-cigarette use was independently associated with progression to heaving patterns of cigarette smoking among US adolescents [80]. Second, we do not know the type of e-cigarette currently used by cigarette-smoking adults. Second generation e-cigarettes (e.g., tank-style systems) deliver nicotine more efficiently than the first generation e-cigarettes used in Bullen et al. trial [49,81]. Third generation e-cigarettes (e.g., advanced personal vaporizers) deliver nicotine at approximately the same level and speed as traditional cigarettes [82]. However, we do not yet know the national prevalence of second and third generation e-cigarette use among current cigarette smokers who are trying to quit, and no published trials or cohort studies estimate cessation efficacy or effectiveness of third-generation e-cigarettes.

Third, in our calculation of benefit, we did not consider the possibility that e-cigarette use among current cigarette smokers leads to a reduction in the intensity of cigarettes smoked per day. A trial conducted by Caponnetto et al. found e-cigarette reduced the median number of cigarettes smoked per day among 300 Italian smokers not intending to quit [83]. Yet, similar reductions in the number of cigarettes smoked per day has not been observed in the US between dual users of e-cigarettes and cigarettes and exclusive cigarette smokers [65].

Fourth, we did not consider the potential population-level health benefit or harm of e-cigarette use among former cigarette smokers because no published trials or cohort studies assessed whether e-cigarette use among former cigarette smokers led to higher or lower rates of relapse to cigarette smoking. A recent cross-sectional study suggested long-term former cigarette smokers who use e-cigarettes may not experience any higher rate of relapse to smoking than their counterparts who do not use e-cigarettes [84].

Current public health models may yield substantively different conclusions about the net harm or benefit of e-cigarette use because there is insufficient data on the effect of e-cigarette use on cigarette smoking-related transitions and tobacco-related diseases. Conclusions may also differ because of decisions—both implicit and explicit—about the framework and underlying assumptions inherent in the model. The host of decisions required to develop a model produce structural uncertainty that may exceed parameter uncertainty [85,86]. Sensitivity analysis will not capture structural uncertainty because the model, itself, remains constant. Future work could incorporate Bayesian model averaging to account structural, or model-based, uncertainty [87]. Future work could also grade the quality of models based on published best practices [86,88].

In conclusion, based on currently available evidence on the e-cigarette associated transition probabilities of cigarette smoking cessation and initiation, our study suggests that e-cigarettes pose more harm than they confer benefit at the population level. If e-cigarettes are to confer a net population-level benefit in the future, the effectiveness of e-cigarettes as a smoking cessation tool will need to be much higher than it currently is. The US Preventive Services Task Force concludes the existing scientific evidence is insufficient to clinically recommend e-cigarettes as a smoking cessation tool [89]. In the United Kingdom, the National Institute of Clinical Excellence also notes limited evidence on the long-term health effects of e-cigarette use and does not clinically recommend e-cigarettes for smoking cessation, in contrast to Public Health England and the Royal College of Physicians [35,90,91]. Additionally, comprehensive tobacco control efforts are needed to reduce the appeal of e-cigarettes to youth.

## Supporting information

S1 Appendix E-vigarette-associated Δ transition probability of cigarette smoking cessation. (DOCX)

S2 Appendix E-cigarette-associated Δ transition probability of cigarette smoking initiation. (DOCX)

S3 Appendix Model parameters. S3 Appendix including Tables A and B. Table A shows model parameters for current adult cigarette smokers. Table B shows model parameters for adolescents and young adults.
(DOCX)
