# Supplementary material for: Quantifying population-level health benefits and harms of e-cigarette use in the United States
Source: PLoS One. 2018 Mar 14;13(3):e0193328. doi: 10.1371/journal.pone.0193328 (PMC5851558; doi:10.1371/journal.pone.0193328)
Supplement: S1 Appendix — (DOCX) [file pone.0193328.s001.docx]

**S1 Appendix. E-Cigarette-Associated Δ Transition Probability of Cigarette Smoking Cessation**

Let A equal a set of age groups. For age group a ∈ A, let quit_a,aid_ equal the probability of cessation using pharmaceutical aids, quit_a,no aid_ equal the probability of cessation not using pharmaceutical aids, quit_a,e-cig_ equal the probability of cessation using e-cigarettes, quit_a,no e-cig_ equal the probability of cessation not using e-cigarettes, and OR_a_ equal the odds ratio of cessation among current cigarette smokers interested in quitting who used e-cigarettes compared to those who did not use e-cigarettes. Pharmaceutical aids included: nicotine patch; nicotine gum or lozenge; nicotine containing nasal spray or inhaler; prescription pill such as Chantix, Varenicline, Zyban, Bupropion, or Wellbutron.

By definition, the odds ratio, OR_a_, equals the ratio of (1) the ratio of the probability of cessation among current cigarette smokers interested in quitting who used e-cigarettes, $\text{quit}_{\text{a,e-cig}},$ and its complement (2) the ratio of the probability of cessation among current cigarette smokers interested in quitting who did not use e-cigarettes, $\text{quit}_{\text{a,no e-cig}}$ and its complement. Conservatively, we set quit_a,no e-cig_ to equal quit_a,aid_. Then, solving for quit_a,e-cig_:

$$\text{quit}_{\text{a,e-cig}}={\frac{\text{OR}_{a} \times\text{quit}_{\text{a,aid}}}{1-\text{quit}_{\text{a,aid}}+\text{OR}_{a} \times\text{quit}_{\text{a,aid}}}.}$$

We then estimated the difference in the probability of cigarette smoking cessation at 6 months between current e-cigarette users and non-current e-cigarette users as the weighted average of (1) the difference in the probability of cessation using e-cigarettes (quit_a,e-cig_) and the probability of cessation using a pharmaceutical aid (quit_a,aid_) and (2) the difference in the probability of cessation using e-cigarettes (quit_a,e-cig_) and the probability of cessation using no pharmaceutical aid (quit_a,no aid_). The weight for (1) equaled the proportion of current cigarette smokers with a past-year quit attempt who used a pharmaceutical aid (p_a_). The weight for (2) equaled 1 minus the proportion of current cigarette smokers with a past-year quit attempt who used a pharmaceutical aid (1-p_a_).

$$\text{∆ prob. cessation}_{\text{a}}\text{=}\text{p}_{\text{a}}\text{×}\left( \text{quit}_{\text{a,e-cig}}\text{- }\text{quit}_{\text{a,aid}} \right)\text{+}\left( \text{1 - }\text{p}_{\text{a}} \right)\text{×}\left( \text{quit}_{\text{a,e-cig}}\text{- }\text{quit}_{\text{a,no aid}} \right)$$

S1 Table 1 presents parameter values for the estimation of the difference in the probability of cigarette smoking cessation at 6 months between current e-cigarette users and non-current e-cigarette users, Δ probability cessation_a_.

| **S1 Table 1. Parameters for Estimation of Difference In The Transition Probability Of Cigarette Smoking Cessation At 6 Months Between Current E-Cigarette Users And Non-Current E-Cigarette Users (%)** | | | | |
| --- | --- | --- | --- | --- |
| Parameter | Notation | Age Range | Point Estimate  (95% CI) | Source |
| Proportion Of Current Cigarette Smokers With a Past-Year Quit Attempt Who Used A Pharmaceutical Aid During Quit Attempt^*^ (%) | p_a_ | 25-34 | 25.9 (21.5, 30.2) | 2010 NHIS |
|  |  | 35-49 | 39.5 (35.0, 44.0) |  |
|  |  | 50-69 | 39.1 (34.4, 43.9) |  |
| Probability of Cigarette Smoking Cessation ≥6 Months Among Current Cigarette Smokers Who Seriously Tried to Quit and Used A Pharmaceutical Aid During Quit Attempt (%) | quit_a, aid_ | 25-34 | 8.1 (5.5, 10.8) | Messer  et al.[1] |
|  |  | 35-49 | 9.3 (7.9, 10.6) |  |
|  |  | 50-69 | 8.3 (6.6, 10.0) |  |
| Probability of Cigarette Smoking Cessation ≥6 Months Among Current Cigarette Smokers Who Seriously Tried to Quit and Did Not Use A Pharmaceutical Aid During Quit Attempt (%) | quit_a, no aid_ | 25-34 | 7.9 (6.9, 8.9) | Messer  et al.[1] |
|  |  | 35-49 | 5.2 (4.5, 5.8) |  |
|  |  | 50-69 | 6.4 (5.5, 7.3) |  |
| Odds Ratio of Quitting Smoking Among Smokers with an Interest in Quitting | OR_a_ | 25-69 | 0.86 (0.60, 1.23) | Kalkhoran & Glantz [2] |

Note: CI=confidence interval; OR=odds ratio.

Finally, we estimated the variance of Δ probability cessation_a_ by performing the bootstrap method N=100,000 times. S1 Table 2 shows the point estimates and 95% confidence intervals of Δ probability cessation_a._

| **S1 Table 2. Age-Group-Specific Point Estimate and 95% CI of Δ Probability Cessation (%)** | | | |
| --- | --- | --- | --- |
| Parameter | Notation | Age Range | Point Estimate  (95% CI) |
| Difference In The Transition Probability Of Cigarette Smoking Cessation At 6 Months Between Current E-Cigarette Users And Non-Current E-Cigarette Users (%) | Δ prob. cessation_a_ | 25-34 | -0.92 (-3.73, 2.27) |
|  |  | 35-49 | 1.26 (-1.58, 4.11) |
|  |  | 50-69 | 0.05 (-2.55, 2.77) |

**References**

1. Messer K, Trinidad DR, Al-Delaimy WK, Pierce JP. Smoking Cessation Rates in the United States: A Comparison of Young Adult and Older Smokers. Am J Public Health. 2008;98: 317–322. doi:10.2105/AJPH.2007.112060

2. Kalkhoran S, Glantz SA. Modeling the Health Effects of Expanding e-Cigarette Sales in the United States and United Kingdom: A Monte Carlo Analysis. JAMA Intern Med. 2015;175: 1671–1680. doi:10.1001/jamainternmed.2015.4209
