# Supplementary material for: Quantifying population-level health benefits and harms of e-cigarette use in the United States
Source: PLoS One. 2018 Mar 14;13(3):e0193328. doi: 10.1371/journal.pone.0193328 (PMC5851558; doi:10.1371/journal.pone.0193328)
Supplement: S2 Appendix — (DOCX) [file pone.0193328.s002.docx]

**S2 Appendix. E-Cigarette-Associated Δ Transition Probability of Cigarette Smoking Initiation**

The cigarette smoking initiation rate in 2006 equaled 6.6% for 12-17 year olds (95% CI 6.1% to 7.1%) and 8.7% for 18-25 year olds (95% CI 7.9% to 9.5%) based on National Survey on Drug Use and Health (NSDUH) data [1]. We selected 2006 because it was the last year before the introduction of e-cigarettes in the US. We assumed the cigarette smoking initiation rates among never e-cigarette users in 2014 equaled these values in 2006. Next, we utilized the meta-analysis results of Soneji et al. [2] that pooled data from seven cohort studies of e-cigarette associated cigarette smoking initiation [3–9] and fit a random-effects meta-analysis model (S2 Figure). Each of the cohort studies estimated the odds ratio of cigarette smoking initiation for ever e-cigarette users, compared to never e-cigarette users, adjusting for demographic, psychosocial, and behavioral risk factors. These factors — correlated with e-cigarette use and with cigarette use — included age, sex, race/ethnicity, parental educational level, parental cigarette smoking, cigarette smoking among friends, sensation-seeking tendency, depressive symptoms, impulsivity, rebelliousness, parental support, and susceptibility to cigarette smoking. The pooled adjusted odds ratio equaled 3.50 (95% CI 2.38 to 5.16). We estimated cigarette smoking initiation rate among ever e-cigarette users based on this pooled odds ratio and the initiation rate among never e-cigarette user.

Let OR equal the pooled odds ratio, p_1_ equal probability of cigarette smoking initiation among ever e-cigarette users, and p_2_ equal the probability of cigarette smoking initiation among never e-cigarette users. By definition, the odds ratio, OR, equals the ratio of (1) the odds of cigarette smoking initiation among ever e-cigarette users, $\frac{\text{p}_{\text{1}}}{(\text{1-}\text{p}_{\text{1}})}$ and (2) the odds of cigarette smoking initiation among never e-cigarette users, $\frac{\text{p}_{\text{2}}}{(\text{1-}\text{p}_{\text{2}})}$. Then,

$$\text{p}_{\text{1}}\text{=}\frac{\text{OR ×}\frac{\text{p}_{\text{2}}}{\left( \text{1-}\text{p}_{\text{2}} \right)}}{\text{1+OR ×}\frac{\text{p}_{\text{2}}}{\left( \text{1-}\text{p}_{\text{2}} \right)}}.$$

We then apply the above formula by age group and set the cigarette smoking initiation rate of 18-25 year olds to 26-29 year olds, both for never e-cigarette users and ever e-cigarette users.

S2 Table shows the e-cigarette associated Δ transition probability of cigarette smoking initiation, which is defined as the difference in the probability of cigarette smoking initiation between ever e-cigarette users and never e-cigarette users by age group. We estimated the variance of Δ probability initiation by performing the bootstrap method N=100,000 times.

**S2 Figure. Odds ratio of cigarette smoking initiation among ever e-cigarette users compared to never e-cigarette users**

Note: CI=Confidence Interval. The size of the point estimate for each study (blue square) is proportional to the weight of the study in the random effects meta-analysis model. The weight of the study is determined by its inverse variance. Source: Soneji et al. [2].

| **S2 Table. Age-Group-Specific Point Estimate and 95% CI for the Δ Transition Probability of Cigarette Smoking Initiation, Ever E-Cigarette Users Versus Never E-Cigarette Users (%)** | | | |
| --- | --- | --- | --- |
| Age Range | Probability of Cigarette Smoking Initiation Among Never E-Cigarette Users (%) and 95% CI | Probability of Cigarette Smoking Initiation Among Ever E-Cigarette Users (%) and 95% CI | E-Cigarette Associated Δ Transition Probability of Cigarette Smoking  (%) and 95% CI |
| 12-17 | 6.6 (6.1, 7.1) | 20.0 (13.8, 27.3) | 13.41 (7.73, 20.15) |
| 18-29 | 8.7 (7.9, 9.5) | 25.2 (17.6, 33.8) | 16.48 (9.68, 24.34) |

**References**

1. U.S. Department of Health and Human Services. Preventing tobacco use among youth and young adults: a report of the Surgeon General. Atlanta, GA: U.S. Department of Health and Human Services, Centers for Disease Control and Prevention, National Center for Chronic Disease Prevention and Health Promotion, Office on Smoking and Health; 2012.

2. Soneji S, Barrington-Trimis JL, Wills TA, Leventhal AM, Unger JB, Gibson LA, et al. Association Between Initial Use of e-Cigarettes and Subsequent Cigarette Smoking Among Adolescents and Young Adults: A Systematic Review and Meta-analysis. JAMA Pediatr. 2017;171: 788–797. doi:10.1001/jamapediatrics.2017.1488

3. Leventhal AM, Strong DR, Kirkpatrick MG, et al. Association of electronic cigarette use with initiation of combustible tobacco product smoking in early adolescence. JAMA. 2015;314: 700–707. doi:10.1001/jama.2015.8950

4. Primack BA, Soneji S, Stoolmiller M, Fine MJ, Sargent JD. Progression to traditional cigarette smoking after electronic cigarette use among US adolescents and young adults. JAMA Pediatr. 2015; 1–7. doi:10.1001/jamapediatrics.2015.1742

5. Wills TA, Knight R, Sargent JD, Gibbons FX, Pagano I, Williams RJ. Longitudinal study of e-cigarette use and onset of cigarette smoking among high school students in Hawaii. Tob Control. 2016; 1–6. doi:10.1136/tobaccocontrol-2015-052705

6. Barrington-Trimis JL, Urman R, Berhane K, Unger JB, Cruz TB, Pentz MA, et al. E-Cigarettes and Future Cigarette Use. Pediatrics. 2016; e20160379. doi:10.1542/peds.2016-0379

7. Brian Primack, Ariel Shensa, Jaime E. Sidani, Beth L. Hoffman, Samir Soneji, Michael J. Fine, et al. Initiation of Cigarette Smoking After E-Cigarette Use: A Nationally Representative Study. Washington DC; 2016. Available: http://www.sbm.org/UserFiles/file/am16-final_v15-lores_forwebsite.pdf

8. Spindle TR, Hiler MM, Cooke ME, Eissenberg T, Kendler KS, Dick DM. Electronic cigarette use and uptake of cigarette smoking: A longitudinal examination of U.S. college students. Addict Behav. 2016;67: 66–72. doi:10.1016/j.addbeh.2016.12.009

9. Richard Miech, Megan E. Patrick, Patrick M. O’Malley, Lloyd Johnston. E-cigarette use as a predictor of cigarette smoking: results from a 1-year follow-up of a national sample of 12th grade students. Tob Control. 2017;
