# Supplementary material for: Quantifying population-level health benefits and harms of e-cigarette use in the United States
Source: PLoS One. 2018 Mar 14;13(3):e0193328. doi: 10.1371/journal.pone.0193328 (PMC5851558; doi:10.1371/journal.pone.0193328)
Supplement: S3 Appendix — including Tables A and B. Table A shows model parameters for current adult cigarette smokers. Table B shows model parameters for adolescents and young adults. (DOCX) [file pone.0193328.s003.docx]

**S3 Appendix. Model Parameters**

Table A shows the value of each model parameter used to estimate the number of years of life gained or lost among the additional number of current cigarette smokers who quit smoking through the use of e-cigarettes, compared to those who did not use e-cigarettes, and remain continually abstinent from smoking for ≥11 years. Table B shows the value of each model parameter for estimating the number of years of life gained or lost among the additional number of never-cigarette smoking adolescents and young adults who eventually become current daily cigarette smokers (and also smoked 100 cigarettes in lifetime) at age 35-39 through the use of e-cigarettes.

|  | Table A. Model Parameters for Current Adult Cigarette Smokers: Point Estimates and 95% Confidence Intervals | | | | | | | | |
| --- | --- | --- | --- | --- | --- | --- | --- | --- | --- |
| Age Group (Yrs.) | Popl.^a^ | Current  Cig.  Smoking^b^ (%) | Tried to  Quit Within  Past Yr.^c^ (%) | Current E-Cig. Use Among Current Cig. Smokers Who Tried to Quit in Past Yr.^d^  (%) | ∆ Transition Prob. 6-Month Smoking Cessation, Current E-Cig. User Vs. Non-Current E-Cig. User^e^  (%) | Prob. 1 Yr.- Smoking Cessation Given 6 Months- Smoking Cessation^f^  (%) | Prob. ≥6 Yr.- Smoking Cessation Given 1 Yr.- Smoking Cessation^g^ (%) | Relative Harm Reduction of E-Cigarette Use Compared to Cigarette Smoking ^h^ (%) | Yrs. Of  Life  Gained^i^ |
| 25-29 | 21,693,805 | 22.4  (17.6, 27.2) | 48.5  (36.1, 61.0) | 28.8  (13.6, 44.0) | -0.92  (-3.73, 2.27) | 74.0  (70.0, 78.0) | 65.8  (63.1, 68.5) | 95.0  (94.0, 96.0) | 10.0  (8.0, 12.0) |
| 30-34 | 21,327,462 | 21.1  (19.0, 23.2) | 52.0  (46.4, 57.6) | 19.4  (15.3, 23.5) | -0.92  (-3.73, 2.27) | 74.0  (70.0, 78.0) | 65.8  (63.1, 68.5) | 95.0  (94.0, 96.0) | 10.0  (8.0, 12.0) |
| 35-39 | 19,706,638 | 19.5  (17.5, 21.4) | 55.0  (49.3, 60.8) | 19.4  (15.3, 23.5) | 1.26  (-1.58, 4.11) | 74.0  (70.0, 78.0) | 65.8  (63.1, 68.5) | 95.0  (94.0, 96.0) | 9.0  (7.2, 10.8) |
| 40-44 | 20,672,183 | 19.3  (17.5, 21.2) | 48.8  (43.5, 54.1) | 19.6  (15.2, 24.1) | 1.26  (-1.58, 4.11) | 74.0  (70.0, 78.0) | 65.8  (63.1, 68.5) | 95.0  (94.0, 96.0) | 9.0  (7.2, 10.8) |
| 45-49 | 21,005,901 | 18.2  (16.3, 20.0) | 44.0  (38.5, 49.5) | 19.6  (15.2, 24.1) | 1.26  (-1.58, 4.11) | 74.0  (70.0, 78.0) | 65.8  (63.1, 68.5) | 95.0  (94.0, 96.0) | 6.0  (4.8, 7.2) |
| 50-54 | 22,533,778 | 20.0  (17.7, 22.3) | 44.2  (37.8, 50.5) | 19.2  (14.8, 23.7) | 0.05  (-2.55, 2.77) | 74.0  (70.0, 78.0) | 65.8  (63.1, 68.5) | 95.0  (94.0, 96.0) | 6.0  (4.8, 7.2) |
| 55-59 | 21,333,043 | 18.4  (16.6, 20.1) | 45.5  (40.4, 50.7) | 19.2  (14.8, 23.7) | 0.05  (-2.55, 2.77) | 74.0  (70.0, 78.0) | 65.8  (63.1, 68.5) | 95.0  (94.0, 96.0) | 4.0  (3.2, 4.8) |
| 60-64 | 18,327,788 | 14.8  (13.1, 16.5) | 50.8  (44.6, 57.1) | 19.0  (12.5, 25.6) | 0.05  (-2.55, 2.77) | 74.0  (70.0, 78.0) | 65.8  (63.1, 68.5) | 95.0  (94.0, 96.0) | 4.0  (3.2, 4.8) |
| 65-69 | 14,960,007 | 12.3  (10.7, 13.9) | 33.0  (26.7, 39.3) | 19.0  (12.5, 25.6) | 0.05  (-2.55, 2.77) | 74.0  (70.0, 78.0) | 65.8  (63.1, 68.5) | 95.0  (94.0, 96.0) | 2.0  (1.6, 2.4) |
|  | Note: ∆ Prob.=Change in Probability; Cig.=Cigarette; E-Cig.=Electronic Cigarette; Popl.=Population; Prob.=Probability; Yrs.=Years. Values in table rounded to nearest tenth and will not, therefore, exactly match point estimates in Section 3. | | | | | | | | |

| Table B. Model Parameters for Adolescents and Young Adults: Point Estimates and 95% Confidence Intervals | | | | | | |
| --- | --- | --- | --- | --- | --- | --- |
| Age (Yrs.) | Popl.^a^ | Never Tried  Cig. Smoking^j^ (%) | Ever Tried  E-Cig.  Among Never  Cig. Smokers^k^ (%) | ∆ Transition Prob. Cig. Smoking Initiation, Ever E-Cig. User Vs. Never E-Cig User^l^  (%) | Prob. of Daily Cigarette Smoking & ≥100 Cig. Smoked in Lifetime by Age of Cig. Smoking Initiation^m^  (%) | Yrs. Of Life Lost at Age 35-39 Yrs.^i^ |
| 12 | 4,113,960 | 97.2 (96.2, 98.1) | 3.5 (2.7, 4.2) | 13.41 (7.73, 20.15) | 42.3 (34.7, 49.9) | 9.0 (7.2, 10.8) |
| 13 | 4,195,245 | 95.3 (94.3, 96.3) | 5.6 (4.4, 6.9) | 13.41 (7.73, 20.15) | 46.1 (38.7, 53.5) | 9.0 (7.2, 10.8) |
| 14 | 4,191,518 | 91.0 (89.5, 92.4) | 7.0 (5.9, 8.1) | 13.41 (7.73, 20.15) | 38.8 (32.3, 45.4) | 9.0 (7.2, 10.8) |
| 15 | 4,156,979 | 84.8 (83.0, 86.6) | 10.3 (8.9, 11.7) | 13.41 (7.73, 20.15) | 35.2 (29.3, 41.1) | 9.0 (7.2, 10.8) |
| 16 | 4,164,736 | 77.5 (75.4, 79.6) | 13.0 (11.0, 15.1) | 13.41 (7.73, 20.15) | 31.9 (26.3, 37.4) | 9.0 (7.2, 10.8) |
| 17 | 4,191,662 | 69.3 (66.8, 71.8) | 13.2 (11.0, 15.5) | 13.41 (7.73, 20.15) | 27.7 (21.0, 34.4) | 9.0 (7.2, 10.8) |
| 18 | 4,261,523 | 62.5 (59.5, 65.5) | 7.4 (4.1, 10.8) | 16.48 (9.68, 24.34) | 26.5 (21.4, 31.5) | 9.0 (7.2, 10.8) |
| 19 | 4,358,928 | 53.6 (50.4, 56.9) | 9.2 (5.0, 13.4) | 16.48 (9.68, 24.34) | 26.5 (18.7, 34.2) | 9.0 (7.2, 10.8) |
| 20 | 4,439,972 | 44.3 (41.1, 47.5) | 10.5 (6.6, 14.4) | 16.48 (9.68, 24.34) | 21.5 (14.5, 28.4) | 9.0 (7.2, 10.8) |
| 21 | 4,536,131 | 43.3 (40.2, 46.4) | 8.1 (5.1, 11.2) | 16.48 (9.68, 24.34) | 24.9 (16.1, 33.8) | 9.0 (7.2, 10.8) |
| 22 | 4,640,778 | 37.7 (35.6, 39.9) | 13.4 (6.8, 20.0) | 16.48 (9.68, 24.34) | 28.4 (17.3, 39.5) | 9.0 (7.2, 10.8) |
| 23 | 4,681,536 | 37.7 (35.6, 39.9) | 10.5 (6.5, 14.6) | 16.48 (9.68, 24.34) | 21.4 (8.9, 34.0) | 9.0 (7.2, 10.8) |
| 24 | 4,586,906 | 34.2 (32.2, 36.3) | 9.7 (5.5, 14.0) | 16.48 (9.68, 24.34) | 27.2 (10.1, 44.3) | 9.0 (7.2, 10.8) |
| 25 | 4,443,541 | 34.2 (32.2, 36.3) | 8.5 (4.9, 12.1) | 16.48 (9.68, 24.34) | 19.2 (9.8, 28.7) | 9.0 (7.2, 10.8) |
| 26 | 4,355,838 | 34.7 (32.7, 36.7) | 8.0 (4.8, 11.2) | 16.48 (9.68, 24.34) | 41.7 (15.4, 68.0) | 9.0 (7.2, 10.8) |
| 27 | 4,330,394 | 34.7 (32.7, 36.7) | 7.4 (4.3, 10.4) | 16.48 (9.68, 24.34) | 44.0 (12.3, 75.8) | 9.0 (7.2, 10.8) |
| 28 | 4,360,105 | 34.7 (32.7, 36.7) | 2.5 (1.0, 4.0) | 16.48 (9.68, 24.34) | 37.4 (3.4, 71.5) | 9.0 (7.2, 10.8) |
| 29 | 4,311,309 | 34.7 (32.7, 36.7) | 5.5 (2.8, 8.2) | 16.48 (9.68, 24.34) | 23.4 (0.0, 54.4) | 9.0 (7.2, 10.8) |
| Note: ∆ Prob.=Change in Probability; Cig.=Cigarette; E-Cig.=Electronic Cigarette; Popl.=Population; Prob.=Probability; Yrs.=Years. Values in table rounded to nearest tenth and will not, therefore, match point estimates in Section 3. The prevalence of never tried cigarette smoking is equal for 22 and 23 year olds, 24 and 25 year olds, and 26-29 year olds because the National Survey on Drug Use and Health aggregates data for these ages. | | | | | | |
